# Evaluation of pharmacological activities, cytotoxicity and phenolic composition of four *Maytenus* species used in southern African traditional medicine to treat intestinal infections and diarrhoeal diseases

**DOI:** 10.1186/1472-6882-13-100

**Published:** 2013-05-11

**Authors:** Aroke Shahid Ahmed, Lyndy J McGaw, Jacobus N Eloff

**Affiliations:** 1 Phytomedicine Programme, Department of Paraclinical Sciences, Faculty of Veterinary Science, University of Pretoria, Private Bag X04, Onderstepoort 0110, South Africa; 2 Permanent address: Federal Institute of Industrial Research, Oshodi, P.M.B 21023, Ikeja, Lagos, Nigeria

**Keywords:** *Maytenus*, Gastrointestinal tract, Infections, Diarrhoea, Cytotoxicity

## Abstract

**Background:**

Microbial infections and resulting inflammation and oxidative stress are common pathogenesis of gastrointestinal tract (GIT) disorders. In South Africa, several species of the genus *Maytenus* are used in traditional medicine to treat various infectious diseases. Most of the previous work on this genus was focused on nonpolar extracts from the root and bark. In this study, leaf extracts of polar extracts of *Maytenus peduncularis*, *Maytenus procumbens*, *Maytenus senegalensis* and *Maytenus undata* were evaluated for antimicrobial, anti-inflammatory and antioxidant activities to determine their efficacy as therapeutic agents in GIT disorders.

**Methods:**

Phenolic-enriched leaf extracts and fractions were prepared by extracting with acidified 70% methanol and solvent-solvent fractionation. The activities of the fractions against *Staphylococcus aureus*, *Pseudomonas aeruginosa*, *Escherichia coli* and *Enterococcus faecalis* as well as clinical isolates of *Aspergillus fumigatus*, *Candida albicans* and *Cryptococcus neoformans* were determined using a serial microplate dilution method. Antioxidant activities were determined using 1,1-diphenyl-2-picrylhydrazyl (DPPH), 2,2'-azinobis(3-ethylbenzthiazoline-6-sulphonic acid) (ABTS), hydroxyl (OH) radical scavenging and linoleic acid peroxidation inhibitory assays. The phenolic composition as well as the cytotoxicity against Vero cell lines of the crude extracts was evaluated using various standard protocols.

**Results:**

The antimicrobial activities were concentrated in the non-polar fractions of hexane, dichloromethane and ethyl acetate (MICs 19–312 μg/ml). The crude extracts and polar fractions (butanol and water) had moderate to poor antimicrobial activity (MICs 312 to above 2500 μg/ml). The crude extracts and polar fractions had good antioxidant activity (EC_50_ values varied from 1.22 to 607 μg/ml, 1.71 to 312 μg/ml and 23 to 284 μg/ml for DPPH, ABTS and OH respectively. Linoleic acid peroxidation inhibition EC_50_ values of the crude extracts ranged between 27 and 39 μg/ml with relatively low toxicity against Vero cell lines (IC_50_ values 87 to 187 μg/ml). Fractionation of a crude extract with low activity could lead to fractions with more potent activity.

**Conclusion:**

This study justifies the traditional use of leaf crude extracts and fractions from these four plants to remedy gastrointestinal disorders resulting from infection, inflammation and oxidative stress complications. The study also provides rationale for the use of leaf extracts with same beneficial effects in place of unsustainable root and bark harvest.

## Background

The gastrointestinal tract (GIT) is a long tube with huge epithelial surface area through which nutrients and fluid passes for digestion and absorption [1]. The basic sequence of GIT is mouth, oesophagus, stomach, duodenum, small intestine, large intestine, rectum, and the anus, where wastes are eliminated [2]. A healthy GIT is permanently populated with a diverse microbial community, termed the microbiota, which is composed of over 300 different species of prokaryotic and eukaryotic microorganisms. The microbiota microbes exist in complex commensal relationships within the host environment, including the mucosa and luminal contents of the digestive tract [3,4]. Infection of the GIT by pathogenic or opportunistic (intestinal microbiota) microbes cause a variety of intestinal disorders. The disorders may lead to nausea, vomiting, diarrhoea, abdominal pain, intestinal inflammation, bloating and flatulence, or systemic diseases [5]. GIT infectious pathogens (bacteria, virus, protozoa and rarely fungi) have a multitude of proven and hypothetical mechanisms through which they can induce various ailments.

Among the GIT disorders, diarrhoea is the most prevalent and dangerous being a major cause of morbidity and mortality worldwide [5]. Diarrhoea occurs when secretory processes exceed the absorptive capacity of the GIT through four identifiable mechanisms: (1) increased secretion from mucosal; (2) decreased absorption (ions and/or solutes and water); (3) altered motility; and (4) increased permeability [6]. The flushing action of diarrhoea can be beneficial as it leads to removal of harmful luminal contents, however, the resulting dehydration from uncontrolled episode can become life threatening [4]. Mortality from infectious diseases in developed countries, where there are low records of invasive infection is low while in resource-constrained developing countries with more cases of infective ailments, the death rate is high [7]. High prevalence of infectious diarrhoea in resource-constrained countries where most people with immunodeficiency challenges live is associated with high poverty, illiteracy level, lack of portable water, poor sanitation, unhygienic conditions, and inadequate control of vectors and infection of reservoirs [8]. Diarrhoea caused by a variety of definable pathogens (opportunistic infection) is also a common problem in human immunodeficiency virus (HIV)-infected patients with CD4+ counts <200 cells/μl or patients with advanced human immunodeficiency virus (AIDS) [9]. The emergence multi-drug resistance strains to antibiotics are compounding the problems of treatment of infectious diseases in immunocompromised patients [10].

A harmful stimulus in GIT including endotoxins, infections and cytotoxins stimulates macrophages and monocytes to secrete several pro-inflammatory cytokines such as tumour necrosis factor-α, interleukin-1, and interleukin-6 [11]. Nonspecific inflammation induced by these irritants can cause intestinal fluid accumulation which may result in inflammatory diarrhoea [4]. Cytotoxic effects on GIT may lead to villus atrophy, decrease in the digestive and absorption capacities of the intestine, generating a malabsorption components. Microbial fermentation of undigested and non-absorbed nutrients reaching the colon, leads to accumulation of osmotically active solutes causing osmotic diarrhoea [7]. Inflamed GIT lead to persistent changes in enteric nervous system, the intrinsic innervation of the bowel which controls virtually all intestinal functions (motility, secretion, blood flow, mucosal growth and aspects of the local immune system), and smooth muscle function. The resultant colonic dysmotility, hypersensitivity, and dysfunction accompany GIT inflammation give rise to diarrhea, cramping, and pain [7].

Cytokines from activate inflammatory cells can release large amounts of toxic oxygen (peroxide anion, hydrogen peroxide and hypochlorous acid) and nitrogen species, proteases, arachidonic acid metabolites (prostaglandins from cyclooxygenase pathways and leukotrienes from lipoxygenase). The processes cause cellular injury by several mechanisms including peroxidation of membrane lipids and oxidative damage of proteins or DNA. The mechanisms of lipid peroxidation involved free radical or other reactive oxygen species chain reactions to produce toxic products which attack and damage biological molecules [11]. Oxidative species are not dormant products of inflammation, but active molecules in the pathogenesis of inflammatory processes. Gastrointestinal infections are potent stimuli of intestinal membrane lipids peroxidation as an important pathogenic event in infectious diarrhoea [12]. Increased concentrations of reactive species and depleted inherent antioxidant defences in intestinal mucosa are involved in a cycle of infection, malabsorption, and malnutrition [13]. Superoxide dismutase and catalase are endogenous antiperoxidative enzymes that protect the cellular constituents against oxidative damage. However, the efficacy of the endogenous antioxidant defence system may be impaired during inflammation [14]. High antioxidative activity and efficient inhibition of polyunsaturated fatty acid peroxidation of GIT epithelial lipid by pharmacological agents including phytochemicals can significantly counteracted oxidative stress and inflammation in this common and chronic infectious disease.

Treatment of infectious diarrhoea typically targets the specific pathogen causing the condition. Some therapies that may be used include antimicrobial agents, such as ciprofloxacin (bacterial pathogens), such as amphotericin, fluconazole (fungal pathogens) or antiprotozoal agents, such as metronidazole (*Giardia* infection) or albendazole (*Encephalitozoon intestinalis*). Causal pathogens of GIT disorders are becoming resistant to drugs [15-17]. These resistant pathogens have given rise to:

● Infections that would otherwise not have occurred.

● Increased frequency of treatment failures.

● Increased severity of infections [18].

Hence the search for more safe and effective antimicrobial compounds is important. Plant-based medicines have been used for thousands of years in traditional systems of health care to treat a wide range of ailments caused by microbial infections and resultant inflammatory/oxidative stress complications. Search for plants with broad pharmacological activities, but of low toxicity has increasingly gained importance in recent years. In southern Africa, different plants species belonging to the genus *Maytenus* are extensively used in traditional medicines to treat diarrhoea, stomach infections, pain and inflammation of the digestive system, stomach cleansing, chest pain, and skin diseases. The species include *Maytenus accuminata* (L.f.) Loes [19], *Maytenus heterophylla* (Eckl. & Zeyh) Robson [20], *Maytenus peduncularis* (Sond) Loes [21], *Maytenus procumbens* (L.f.) Loes [22], *Maytenus undata* (Thunb) Blakelock [23] and *Maytenus senegalensis* (Lam.) Exell (syn *Gymnosporia senegalensis* (Lam.) Loes) [24-26].

Another important point to consider is that the use of the root or bark stem of trees as medicinal component could compromise the population of the native species. Continuous exploitation of root or bark stem of trees is not sustainable because removal of such parts leads to plant death [23,27]. It is important to verify whether other parts of the plant can have the same beneficial effects with lower toxicity. From the literature, root bark and stem bark of *Maytenus* are primarily used in traditional medicines and the major focus of previous research on the genus. However, there are reports of various uses for the leaves [23,27]. In the Phytomedicine Programme we evaluated and determined the biological activities of tree leaf extracts for medicinal purposes because of the sustainability of leaf harvest compared to roots and stems.

In this study, we hypothesised that phytochemicals from the leaf extracts of *M. peduncularis, M. procumbens*, *M, undata* and *M. senegalensis* may not be be cytotoxic and can provide protection against some enteroinvasive pathogens as well as sustain recovery of the epithelium by promoting inflammatory and oxidative stress healing in infectious diarrhoea. We investigated the rationale for the use of the crude leaf extracts and fractions of various polarities from the four *Maytenus* species as therapeutic and preventative agents, at least in infectious diarrhoea, and perhaps also in diarrhoea associated with acute or chronic inflammation/oxidative damage of the gut.

## Methods

### Plant collection

The leaves of *M. peduncularis*, *M. procumbens*, *M. senegalensis* (syn *Gymnosporia senegalensis* (Lam.) Loes) and *M. undata* were collected by courtesy of Mrs Lita Pauw from the samples kept in the Phytomedicine Programme plant collection. The plant material was originally identified and authenticated by Ms. Lorraine Middleton and Magda Nel at the University of Pretoria Botanical Garden. The voucher specimen numbers in the HWG Schweikert Herbarium were PRU 76382, PRU 77119, PRU 114717, and PRU 18576 for *M. peduncularis*, *M. procumbens*, *M. senegalensis* (syn *Gymnosporia senegalensis*) and *M undata* respectively.

### Preparation of extracts

**Figure 1:**
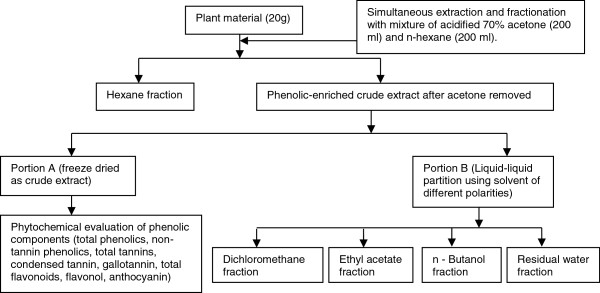
Optimized phenolic-enriched extraction process, fractionation and, analysis of the extract phenolic content.

The extraction process to obtain crude extracts and fractions with varying polarities is presented in Figure 1 as described by Naczk and Shahidi [28] with some modifications. The ground leaf materials (20 g) were extracted with mixture of acidified 70% acetone and n-hexane. The dried polar samples (crude extracts, butanol and residual fractions) were dissolved in 70% acetone or 25% methanol in acetone solution while the non-polar (hexane, ethyl acetate, and dichloromethane) fractions were dissolved in acetone for bioassays and phytochemical analysis.

### Antimicrobial assays

#### Microbial strains

Four standard bacterial strains of the most important nosocomial pathogens including two Gram positive species (*Staphylococcus aureus* (ATCC 29213), *Enterococcus faecalis* (ATCC 29212)), and two Gram negative (*Escherichia coli* (ATCC 25922), *Pseudomonas aeruginosa* (ATCC 27853) were used in this work. The bacteria are commonly involved in GIT infections with resultant inflammation and diarrhoeal symptoms [29-31]. Three pathogenic and opportunistic fungal isolates were obtained from the Bacteriology Laboratory, Department of Veterinary Tropical Diseases, Faculty of Veterinary Science, University of Pretoria and used as test organisms. These fungi represent the different morphological forms of fungi, namely yeasts (*Candida albicans* isolated from a Goldian finch and *Cryptococcus neoformans* isolated from a cheetah) and moulds (*Aspergillus fumigatus* isolated from a chicken), all of which suffered from a systemic mycosis. These fungi are the most common and important opportunistic disease-causing fungi of animals mainly urogenital, respiratory and gastrointestinal complications [32-37].

#### Minimum inhibitory concentration (MIC)

Minimum inhibitory concentration (MIC), a quantitative end point measurement of the concentration of crude leaf extracts (acidified 70% acetone) and fractions of the 4 medicinal plants that halt microbial growth under visible threshold after 18 to 24 h were evaluated [38]. The assay was carried out by broth serial micro dilution assay using 96-well microtitre plate with p-iodonitrotetrazolium violet (INT) added as growth indicator [39].

For antibacterial test, bacterial cultures (*Staphylococcus aureus*, 2.6 × 10^12^ cfu/ml; *Enterococcus faecalis*, 1.5 × 10^10^ cfu/ml; *Pseudomonas aeruginosa*, 5.2 × 10^13^ cfu/ml; *Escherichia coli*, 3.0 × 10^11^ cfu/m were sub-cultured from Mueller Hinton (MH) agar plates with a 10% inoculum inMueller Hinton broth (MHB) (bacteria) was grown overnight. Microtitre plates were prepared by addition of 100 μl of distilled sterilized water to each well. A 100 μl aliquot of the test samples at a starting concentration of 10 mg/ml, positive controls (gentamicin) and negative control (acetone) were added to the first well followed by two fold serial dilutions. The final concentration of test samples was 2500 μg/ml (first well) and 19 μg/ml (last well). To each well, 100 μl of the bacterial cultures was added. This 50% inoculum ensyured there was no lag phase in the growth of the microorganism (39). The plates were airtight sealed and incubated at 37°C under 100% relative humidity conditions overnight. Thereafter, 40 μl of 0.2 mg/ml INT solution was added to all inoculated wells to determine growth inhibition of the microbes. The bacterial growth inhibitions were observed for 2 h at 30 min intervals after the addition of INT [39].

For antifungal test, fungal cultures (*Candida albicans*, 2.5 × 10^6^ cfu/ml; *Aspergillus fumigatus*, 8.1 × 10^6^ cfu/ml and *Cryptococcus neoformans*, 2.6 × 10^6^ cfu/ml) were sub-cultured from Sabouraud agar plates and with a 10% inoculum in Sabouraud dextrose broth were grown overnight. The MICs determined using the serial dilution assay [40] with some modifications involving the addition of INT to the inoculated wells immediately because of the slower growth nature of fungi [41]. Amphoteric B and acetone were used as positive and negative control respectively. The plates were airtight sealed and incubated at 37°C for 36 h after which the fungal growth inhibitions observed for 6 h at 1 h intervals.

#### Antioxidative assays

##### 2, 2-Diphenyl-1-picrylhydrazyl (DPPH) radical scavenging assay

The scavenging activity of crude extracts and fractions was assessed as described by Brand-Williams *et al*., [40] based on the reduction of methanolic DPPH in presence of a hydrogen donating antioxidant with some modifications to 96-well microtitre plate. DPPH, a deep violet coloured solution which has maximal absorption at 516 nm changes to yellow on reduction. The free radical scavenger activity of the compound is proportional to the colour change of DPPH. The methanolic solution of DPPH (0.4 mg/ml, 160 μl) was dispensed from stock and added to 40 μl ascorbic acid and trolox, 1.0–200 μg/ml (positive controls), different concentrations of crude extracts and fractions (3.9–500 μg/ml) or vehicle (an equivalent amount of methanol in the absence of substance test). Thirty minutes later the absorbance was measured at 516 nm using a Versamax microplate reader. The analysis was carried out in triplicate, and the results were expressed as percentage reduction of the initial DPPH absorption in relation to the control group. The concentration of extract that reduced DPPH colour by 50% (EC_50_) was determined.

#### 2, 2’-azinobis (3-ethylbenzthiazoline-6-sulphonic acid) (ABTS) radical scavenging assay

The ABTS radical scavenging capacity of the samples was measured with modification to a 96-well microtitre plate format [42]. ABTS radical was generated by reacting 7 mM solution of ABTS and 2.45 mM solution of potassium persulfate at room temperature for 12 h. The ABTS radical stock solution was adjusted to 7.00 ± 0.02 at 734 nm before use. The test samples (40 μl) were made in a concentration range of 0.3-250 μg/ml by two fold serial dilutions and 160 μl of ABTS radical solution was added. Absorbance was measured after 6 min at 734 nm. Trolox and ascorbic acid were used as positive controls, methanol as negative control and extract without ABTS as blank.

#### Hydroxyl radical scavenging assay

The hydroxyl radical scavenging activity of the extracts was determined with some modifications [43]. The hydroxyl radical was generated by using the Fenton reaction where 50 ml of ferrous chloride (8.0 mM), 80 ml of hydrogen peroxide and 50 ml of distilled water, was allowed to stand for 1 h. The mixture was filtered to remove the debris. Hydroxyl radical was determined by mixing 120 μl of the hydroxyl radical solution with 66 μl of the extracts followed by 14 μl of salicylic acid (20 mM). The mixture was incubated for 30 min at 37°C and absorbance read at 510 nm.

#### Linoleic acid peroxidation inhibitory assay

The inhibition by the extracts of lipid peroxidation was determined according to the thiobarbituric acid method [44] with some modifications. The Fenton reaction (Fe^2+^+ H_2_O_2_ → Fe^3+^+OH^·^) was used to induce the linoleic acid emulsion peroxidation [45]. In this assay, the aliquot contain 2.5 ml of linoleic acid solution in methanol, 150 μl of TRIS HCl, 50 μl of ascorbic acid, 500 μl of extract (8.0-250 μg/ml) and 50 μl of FeSO_4_. The mixture was incubated at 37°C for 60 min in the dark and the reaction was terminated by adding 516 μl of 40% trichloroacetic acid dissolved in 0.01% sodium hydroxide solution. The MDA generated was measured by adding 1.6 ml of thiobarbituric acid solution and the reaction mixture heated at 95°C for 15 min. The absorbance was recorded at 510 nm.

#### Cytotoxicity assay of the crude extracts against Vero cell lines

Cytotoxicity of the extract was determined by the MTT [3-(4, 5-dimethylthiazol-2-yl)-2, 5 diphenyltetrazolium bromide] assay [46] using a Vero African green monkey kidney cell line. The cells were cultured in Minimal Essential Medium (MEM) Earle’s Base (Sigma), supplemented with 2 mM L-glutamine, 16.5 mM NaHCO_3_ together with 0.1% gentamicin (Virbac) and 5% foetal calf serum (Sigma). Confluent monolayer culture suspensions of the cells were seeded into 96-well tissue culture microtitre plates at a density of 2 × 10^3^ cells per well and incubated for 24 h at 37°C in a 5% CO_2_ incubator. The MEM was removed from the cells, and extracts at various concentrations, positive control (berberine chloride (Sigma)) and negative controls were added and incubated for 5 days. The cells were observed using an inverted microscope to check for cytopathic effect from the extract. The cell proliferation and viability was quantified by addition of 30 μl of a 5 mg/ml solution of MTT in PBS to each well and incubation for another 4 h at 37°C. The medium was carefully removed from the wells without disturbing the MTT concentrate and washed twice with PBS. The liquid was aspirated from the cells and 50 μl of DMSO was added to each well to dissolve the crystallized MTT formazan. The amount of reduced MTT was measured as absorbance at 570 nm using a Versamax microtitre plate reader. The results were expressed as a percentage growth of the control cells and IC_50_ values were calculated (Table 1).

**Table 1:** Cytotoxicity of the acidified 70% acetone extracts

**Test samples**	**Cytotoxicity (IC**_**50 **_**(μg/ml))**	**Lipid peroxidation inhibition (LC**_**50 **_**(μg/ml))**	**Lipoxygenase inhibition (LL**_**50 **_**(μg/ml)**
*Maytenus peduncularis*	89.41 ± 16.6	39.84 ± 5.52	44.15 ± 5.60
*Maytenus procumbens*	187.71 ± 19.92	34.21 ± 1.63	117.93 ± 36.50
*Maytenus senegalensis*	87.62 ± 3.02	27.21 ± 2.3	115.80 ± 20.11
*Maytenus undata*	99.17 ± 11.88	33.70 ± 0.85	50.92 ± 25.70
Berberine (positive control)	4.74 ± 0.41		

#### Phytochemical analysis

##### Determination of total phenolic constituents of the extracts

The total phenolic constituents of the extracts were determined using Folin-Ciocalteau method with some modifications [47]. The crude extracts (50 μl) at concentration of 1:1 (mg:ml/w:v) plant material and extracting solvent was mixed with 500 μl distilled water, 250 μl of commercial Folin-Ciocalteau reagent diluted with distilled water (1:1) and 1250 μl of 20% sodium carbonate solution. Absorbance of the mixture was recorded at 725 nm after incubation for 40 min. The concentration of polyphenols (expressed as mg Gallic /g dry weight) was calculated from a linear equation of the standard curve (0.0019-0.25 mg/ml gallic acid) prepared at the same time using the following equation:

$$\begin{array}{l} \text{Absorbance}=4.9022\;\times \;\mathrm{TP}\left(\mathrm{mg}\;\mathrm{GAE}\right),\;R^{2}\\ \;=0.9804 \end{array}$$

#### Determination of total tannin content

The total tannin content of the extracts was determined by using the polyvinylpyrrolidone (PVPP) binding method [47]. The tannin-binding mixtures were prepared by mixing 100 mg of PVPP, 1.0 ml of distilled water and 1.0 ml of tannin-containing extracts in a centrifuge tube. The mixtures were thoroughly mixed by vortexing and kept at 4°C for 15 min. The mixture was thawed and filtered using Whatman number 1 filter paper to remove the bound tannin. The filtrate (100 μl) was dispensed into a test tube and the phenolic content determined as described above. Non-tannin phenolic composition was determined from the standard curve of catechin expressed as catechin equivalent in mg/g dry material. The tannin content was calculated as the difference between the total phenolic and non-phenolic content of the extracts because the tannin was bound and precipitated by PVPP.

#### Determination of proanthocyanidin content

The proanthocyanidin content of the extracts was determined using the butanol-HCl assay [47]. The extract (500 μl) was dispensed into a test tube and diluted to 10 ml with 70% acetone. 3.0 ml of 95/5 butanol/HCl reagent and 100 μl of 2% ferric ammonium sulphate in 2 N HCl were added. The test tubes were loosely covered and heated in a boiling water bath for 50 min. The absorbance was recorded at 550 nm after the tubes and contents were cooled to room temperature. Absorbance of the unheated mixture was used as a blank.

#### Determination of condensed tannin content

The condensed tannin content of the extracts was determined using the vanillin/HCl assay [48]. To 0.5 ml of the extract measured into a test tube, 3 ml of vanillin reagent containing 4% concentrated HCl and 0.5% of vanillin in methanol was added. The mixture was allowed to stand for 15 min. The absorbance was recorded at 500 nm against methanol as a blank. The concentration of condensed tannin in the extracts expressed as catechin equivalent (CE)/g dry plant material was calculated using the following equation:

$$\begin{array}{l} \text{Absorbance}=0.1791\;\times \;\mathrm{CT}\left(\mathrm{mg}\;\mathrm{CE}\right)+0.0504,\;R^{2}\\ \;=0.9440. \end{array}$$

#### Determination of hydrolysable tannin (gallotannin)

The gallotannin content of the extracts was determined with the potassium iodate assay [49]. To 3 ml of the extract, 1 ml of saturated solution of potassium iodate was added and allowed to stand at room temperature for 40 min. The absorbance was read at 550 nm. A standard curve was prepared using gallic acid under the same conditions as the extracts and results expressed as gallic acid equivalent (GAE)/g dry plant material was calculated using the following equation:

$$\begin{array}{l} \text{Absorbance}=0.8264\;\times \;\mathrm{GT}\left(\mathrm{mg}\;\mathrm{GAE}\right)\\ \;+0.0392,\;R^{2}\\ \;=0.9155. \end{array}$$

#### Determination of total flavonoids and flavonols

The total flavonoid content of the extracts was determined by the aluminium chloride method with some modifications [50]. Briefly 100 μl of the extract was mixed with 100 μl of 20% AlCl_3_ and two drops of glacial acetic acid. The mixture was diluted with methanol to 3000 μl. Absorbance was read at 415 nm after 40 min. Blank samples were prepared with the extract without AlCl_3_. Standard curve was prepared using quercetin (3.9-500 μg/ml) in methanol under the same conditions. The concentration of flavonoids was expressed as mg quercetin equivalent/g of dry plant material was calculated using the following equation:

$$\begin{array}{l} \text{Absorbance}=0.9747\;\times \;\mathrm{FT}\left(\mathrm{mg}\;\text{quercetin}\right){,R}^{2}\\ \;=0.9846. \end{array}$$

The flavonol content of the extracts was determined by the aluminium chloride method [50] with some modifications. Extract (1 ml) was mixed with 1 ml of 20 mg/ml of AlCl_3_ and 3 ml of 50 mg/ml of sodium acetate. A standard curve was prepared using quercetin (0.0019-0.0312 mg/ml) in methanol under the same conditions. Absorbance was read at 440 nm after 2.5 h. The concentration of flavonol was expressed as mg quercetin equivalent/g of dry plant material was calculated using the following equation:

$$\begin{array}{l} \text{Absorbance}=34.046\;\times \;\mathrm{FLL}\left(\mathrm{mg}\;\text{quercetin}\right),\;R^{2}\\ \;=0.9853. \end{array}$$

#### Lipoxygenase inhibitory assay

The soybean 15-lipoxygenase (LOX) enzyme inhibition was measured in borate buffer (0.2 M, pH 9) by following the increase in absorbance at 234 nm from 30 s after addition of the enzyme, using linoleic acid (134 *μ*M) as substrate [51]. The final enzyme concentration was 200 U/ml. Test substances were added as DMSO solutions (final DMSO concentration of 1.6%); DMSO alone was added in control experiments. The enzyme solution was kept on ice, and controls were measured at intervals throughout the experimental periods to ensure that the enzyme activity was constant. All measurements were carried out in triplicate for the controls and each concentration of the test samples. Quercetin was employed as a positive control. Calculation of enzyme activity was carried out [32], and IC_50_ values were determined by non-linear interpolation between the measuring points closest to 50% activity.

#### Statistical Analysis

All values were expressed as mean ± SD. The sigmoidal dose–response (variable slope) best fit EC_50_ for each extract and fraction was computed with the associated standard error. The analysis was performed using GraphPad Prism for Windows, version 5.03 (GraphPad Software, San Diego, CA, USA). Statistical analysis of the results was performed by using one way ANOVA, followed by Bonferroni multiple comparison post hoc tests and the significant difference was set at P < 0.05.

## Results

### Yield of extraction and fractionation

**Figure 2:**
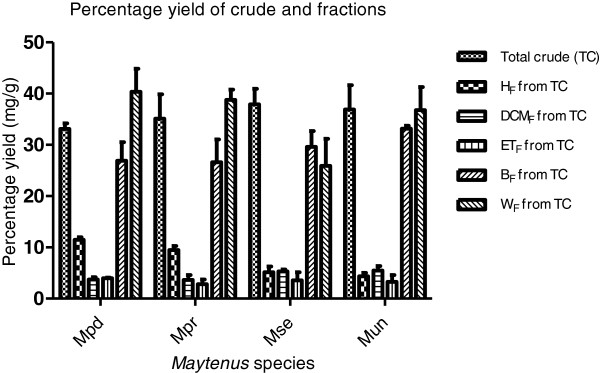
**Yield of crude extract and fractions.** Mpd = *Maytenus peduncularis*, Mpr = *Maytenus procumbens*, Mse = *Maytenus senegalensis*, Mun = *Maytenus undata*, TC = total crude, H_F_ = hexane fraction, DCM_F_ = dichloromethane, ET_F_ = ethyl acetate, B_F_ = butanol fraction, W_F_ = water fraction.

The yield of the extracts and the fractions of various polarities using hexane, dichloromethane, ethyl acetate, butanol and residual water are presented in Figure 2. Maximum yield was obtained for the extracts of *M. senegalensis* (37.89 ± 3.05%) followed by *M. undata* (36.89 ± 4.775), while *M. peduncularis* (33.12 ± 1.07%) gave the lowest yield. The extraction process efficiently removed the chlorophyll from the bulk of the 70% acetone extract into the hexane portion. A part of the dried residual water fraction could not be redissolved due to the formation of insoluble complexes between the polyphenolics and other high molecular weight components such as polysaccharides.

### Antimicrobial activities of the crude extracts and fractions

The minimum inhibitory concentrations (MICs) of the four *Maytenus* phenolic-enriched crude extracts and fractions of various polarities against some enteric pathogens are presented in Table 2. All the crude extracts and fractions of various polarities have antimicrobial activities. Generally for all tested medicinal plants, the crude extracts and polar fractions (butanol and water) were less active than the non-polar fractions (hexane and dichloromethane) while the intermediate polar fraction (ethyl acetate) were moderately active. The hexane and dichloromethane fractions of *M. senegalensis* had good inhibitory activities the bacteria and fungi (MICs varying from 156 to 39 μg/ml). All the bacteria were resistant to water fraction at the highest concentration tested but moderately sensitive to crude extract (MICs ranging from 312to 2500 μg/ml) with *E. coli* and *E. faecalis* being the most resistant (MIC = 2500 μg/ml). The most susceptible bacteria were *P. aeruginosa* to crude extract (625 μg/ml), hexane (39 μg/ml), dichloromethane (78 μg/ml), ethyl acetate (156 μg/ml), Butanol (1250 μg/ml), and *S. aureus* to crude extract (625 μg/ml), hexane (78 μg/ml), dichloromethane (39 μg/ml), ethyl acetate (156 μg/ml), butanol (625 μg/ml).

**Table 2:** Antibacterial and antifungal activities of the crude extracts and the fractions expressed as minimum inhibitory concentration (μg/ml)

	***Maytenus peduncularis***						***Maytenus procumbens***					
Microbial spp	CrE	Hex	DCM	ETOAc	But	Water	CrE	Hex	DCM	ETOAc	But	Water
*E. c*	312	39	39	78	625	>2500	1250	39	39	156	1250	>2500
*E. f*	312	39	39	156	1250	>2500	>2500	39	39	156	625	>2500
*P. a*	312	19	19	156	312	>2500	312	78	39	156	625	>2500
*S. a*	>2500	78	39	312	78	>2500	1250	39	78	312	625	>2500
*C. a*	312	312	78	312	156	312	1250	156	39	156	312	625
*C. n*	78	312	78	78	1250	2500	625	156	39	156	156	2500
*A. f*	312	78	156	156	312	625	1250	78	78	78	156	625
	***Maytenus senegalensis***						***Maytenus undata***					
Microbial spp	CrE	Hex	DCM	ETOAc	But	Water	CE	Hex	DCM	ETOAc	But	Water
*E. c*	2500	78	78	156	1250	>2500	1250	312	156	156	>2500	>2500
*E. f*	2500	39	39	312	625	>2500	312	156	156	312	2500	>2500
*P. a*	625	39	78	156	1250	>2500	312	78	78	78	2500	>2500
*S. a*	625	78	39	156	625	>2500	625	156	156	156	312	>2500
*C. a*	1250	78	39	156	156	625	1250	625	78	312	625	1250
*C. n*	156	156	39	78	78	1250	156	1250	78	78	312	2500
*A. f*	625	156	78	156	1250	312	1250	625	625	156	625	625
	**Controls**											
Microbial spp	Gent	Amp B	70% ace	Distilled Water								
*E. c*	0.39	-	>2500	>2500								
*E. f*	0.78	-	>2500	>2500								
*P. a*	1.56	-	>2500	>2500								
*S. a*	0.78	-	>2500	>2500								
*C. a*	-	6.25	>2500	>2500								
Microbial spp	Gent	Amp B	70% ace	Distilled Water								
*E. c*	0.39	-	>2500	>2500								

N.B: E. c = *Escherichia coli*, E. f = *Enterococcus faecalis*, P. a = *Pseudomonas aeruginosa*, S. a = *Staphylococcus aureus*, C. a = *Candida albicans*, C. n = *Cryptococcus neoformans*, A. f = *Aspergillus fumigatus*, Gent = gentamicin, Amp B = amphotericin B, .CrE = crude extract, Hex = hexane fraction, DCM = dichloromethane, ETOAc = ethyl acetate and But = n- Butanol.

The fungi were moderately sensitive to the crude extract (MICs ranging from 1250 to 156 μg/ml). *C. neoformans* was the most sensitive fungus to dichloromethane, ethyl acetate, butanol (MICs = 78 μg/ml), crude extract, and hexane (MICs = 156 μg/ml). The non-polar fractions of *M. procumbens* had the largest broad spectrum activities as they were active against all the microorganisms investigated with good MIC value (MIC = 39 μg/ml). *E. coli*, *E. faecalis*, *P. aeruginosa*, *C. albicans* and *C. neoformans* (MIC = 39 μg/ml), and *S. aureus* and *A. fumigatus* (MIC = 78 μg/ml) were the most sensitive to the dichloromethane fractions. The organisms were also very sensitive to hexane fractions (*E. coli*, *E. faecalis*, *S. aureus*, (MIC = 39 μg/ml), and *P. aeruginosa* and *A. fumigatus* (MIC = 78 μg/ml)), *C. albicans* and *C. neoformans* (MIC = 156 μg/ml), the crude extract, ethyl acetate and butanol fractions were moderately active (MIC ranging from 1250 to 156 μg/ml) whereas the water fraction was less active (MIC ranging from 312 to >2500 μg/ml). *M. peduncularis* had the highest inhibitory activities obtained with its hexane and dichloromethane fractions on P. aeruginosa (MIC = 19 μg/ml), and on *E. coli* and *E. faecalis* (MIC = 39 μg/ml). The dichloromethane fraction had a broad based spectrum of activity against all tested microorganisms (MICs varying from 156 to 19 μg/ml) with *A. fumigatus* being the least sensitive (MIC = 156 μg/ml). *M. undata* had the least inhibitory activity as all the microorganisms tested have some resistance to crude extracts and various fractions (MICs varying from >2500 to 312 μg/ml) except P. aeruginosa that was sensitive to hexane, dichloromethane, and ethyl acetate (MIC = 78 μg/ml), and *C. neoformans* to dichloromethane and ethyl acetate (MIC = 78 μg/ml). Acetone solution (70%), used to dissolve the plant extracts did not have any inhibitory activity on the microbial growth.

### Antioxidant assays

The phenolic-enriched extracts and fractions of various polarities from the leaves of four *Maytenus* species had strong antioxidant activities determined by the four different methods, namely the DPPH radical, ABTS radical, hydroxyl ion scavenging and linoleic acid peroxidation inhibitory activities Tables 3 and 1. For each sample, eight concentrations between 0.5 and 250 μg/ml were evaluated and the quantifications of the activities were expressed by calculating the EC_50_. The results indicated that the crude extracts of the four plant species have good DPPH scavenging capacity with EC_50_ values of 1.88 ± 0.02, 3.56 ± 0.16, 6.71 ± 0.26 and 3.48 ± 0.09 μg/ml followed by ethyl acetate fractions with EC_50_ values of 2.33 ± 0.21, 1.22 ± 0.15, 9.50 ± 0.11and 4.55 ± 0.31 μg/ml for *M. peduncularis*, *M. procumbens*, *M. senegalensis* and *M. undata* respectively. Hexane and water fractions were inactive with EC_50_ values greater than 110.70 ± 10.69 respectively for all the four plant species. The control compounds were trolox and ascorbic acid with EC_50_ values of.1.18 ± 0.06 and 1.50 ± 0.06 μg/ml respectively. For the ABTS radical decolourization, scavenging patterns were similar to that of DPPH, although the EC_50_ values were comparatively lower in the ABTS assay for all corresponding test samples. Crude extracts of the four plants had good DPPH scavenging activity with EC_50_ values of 8.65 ± 0.13, 4.03 ± 0.19, 5.34 ± 0.39 and 7.89 ± 0.31 μg/ml followed by ethyl acetate fractions with EC_50_ values of 6.34 ± 0.18, 1.71 ± 0.13, 3.59 ± 0.06 and 6.66 ± 1.54 μg/ml for *M. peduncularis*, *M. procumbens*, *M. senegalensis* and *M. undata* respectively. For butanol fractions, the EC_50_ values were as follows: 52.79 ± 14.44, 8.89 ± 2.86, 7.78 ± 3.13, and 5.74 ± 1.37 μg/ml for *M. peduncularis*, *M. procumbens*, *M. senegalensis,* and *M. undata* respectively. . The variation in the ABTS and DPPH radical scavenging activity of the butanol fractions may be due to differences in antiradical mechanisms. The efficiency of an antioxidant component to reduce DPPH largely based on a hydrogen atom mechanism while the ABTS assay is based on an electron transfer mechanism with faster reaction kinetics [52]. The radical scavenging activity of an extract depends on the quality of the active components rather than the quantity, especially the presence of hydroxyl groups and their position.

**Table 3:** **Free radical scavenging activities of the *Maytenus *species expressed as EC**_**50 **_**(μg/ml)**

	***Maytenus peduncularis***						
**Assay**	**Crude**	**Hex**	**DCM**	**ETOAc**	**But**	**Water**	**Trolox**
DPPH	1.88 ± 0.02	113.13 ± 12.63	28.19 ± 4.14	2.33 ± 0.21	59.91 ± 8.01	153.40 ± 20.62	1.18 ± 0.06
ABTS	8.65 ± 0.13	114.64 ± 25.93	33.54 ± 1.29	6.35 ± 0.18	52.79 ± 14.44	74.89 ± 2.84	
OH	23.92 ± 2.28	110.54 ± 17.91	122.07 ± 20.50	70.86 ± 18.09	49.55 ± 5.7		
	***Maytenus procumbens***						
DPPH	3.56 ± 0.16	110.70 ± 10.69	30.67 ± 4.54	1.22 ± 0.15	20.64 ± 0.90	189.00 ± 7.56	1.20 ± 0.06
ABTS	4.03 ± 0.19	277.80 ± 16.14	22.26 ± 1.36	1.71 ± 0.13	8.99 ± 2.86	130.70 ± 15.06	
OH	107.69 ± 12.32	179.70 ± 41.17	223.96 ± 42.04	76.70 ± 11.58	46.79 ± 12.42		
	***Maytenus senegalensis***						
DPPH	6.71 ± 0.26	251.43 ± 29.54	121.27 ± 10.99	9.50 ± 0.11	48.10 ± 0.26	162.17 ± 22.77	1.28 ± 0.07
ABTS	5.34 ± 0.39	312.75 ± 43.83	139.90 ± 13.65	3.59 ± 0.06	7.78 ± 3.13	62.86 ± 3.97	
OH	146.30 ± 21.60	187.40 ± 55.56	356.80 ± 37.39	42.06 ± 12.90	30.81 ± 1.78		
	***Maytenus undata***						
DPPH	3.48 ± 0.07	160.50 ± 31.40	42.91 ± 6.16	4.55 ± 0.31	15.86 ± 0.31	607.30 ± 7.57	1.31 ± 0.07
ABTS	7.89 ± 0.31	268.30 ± 7.78	55.30 ± 5.09	6.66 ± 1.54	5.74 ± 1.37	220.27 ± 30.15	
OH	80.68 ± 2.9	284.36 ± 27.04	311.90 ± 150.33	155.53 ± 35.85	51.19 ± 5.30		

CrE = crude extract, Hex = hexane fraction, DCM = dichloromethane, ETOAc = ethyl acetate and But = n- Butanol, DPPH. = 2, 2-Diphenyl-1-picrylhydrazyl radical, ABTS = 2,2’-azino-bis 3-ethylbenzothiazoline-6-sulfonic acid, OH = hydroxyl radical.

The scavenging capacities of the extracts on hydroxyl radical inhibition by the salicylic acid oxidation method expressed as EC_50_ are presented in Table 3. Crude extract of the four plants have good OH radical scavenging power with EC_50_ values of 23.92 ± 2.29, 107.69 ± 12.32, 146.30 ± 21.60 and 80.68 ± 2.90 μg/ml followed by butanol fractions with EC_50_ values of 49.55 ± 5.70, 46.79 ± 12.42, 30.81 ± 1.78 and 51.19 ± 5.30 μg/ml for *M. peduncularis*, *M. procumbens*, *M. senegalensis* and *M. undata* respectively. For ethyl acetate fractions, the EC_50_ values were as follows: 70.88 ± 18.09, 76.70 ± 11.58, 42.06 ± 12.90, and 155.53 ± 35.85 μg/ml for *M. peduncularis*, *M. procumbens*, *M. senegalensis,* and *M. undata* respectively. All crude leaf extract of the four plant species led to dose-dependent lipid peroxidation of linoleic acid induced by FeSO_4_-H_2_O_2_ system, which was monitored by the generation of malonydialdehyde MDA. The results expressed as EC_50_ are presented in Table 1. The EC_50_ values were 39.84 ± 5.52, 34.21 ± 1.63, 27.21 ± 2.3, and 33.70 ± 0.85 μg/ml for *M. peduncularis*, *M. procumbens*, *M. senegalensis,* and *M. undata* respectively. *M. senegalensis* was the most active while *M. peduncularis* was the least active among the extracts.

### Cytotoxicity

The cytotoxicity of the phenolic-enriched crude extracts of the four *Maytenus* species is presented in Table 1. The results indicate that the extracts are relatively non-toxic to Vero cell lines with IC_50_ values of 89.41 ± 16.80, 187.71 ± 19.92, 87.82 ± 3.02, and 99.17 ± 11.88 μg/ml for *M. peduncularis*, *M. procumbens*, *M. senegalensis,* and *M. undata* respectively. In categorizing plant extract safety, IC_50_ values of 20 μg/ml and below were considered to be toxic [53]. Therefore, all the phenolic-enriched extracts of the four *Maytenus* species showed no preliminary indication of toxicity although *in vivo* studies would need to confirm their safety for use in treating diarrhoea.

### Phenolic constituents

**Figure 3:**
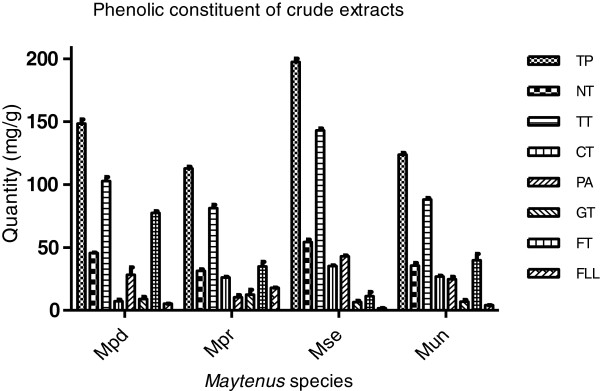
**Phenolic compositions of the acidified 70% acetone leaf extracts.** Mpd = *Maytenus peduncularis*, Mpr = *Maytenus procumbens*, Mse = *Maytenus senegalensis*, Mun = *Maytenus undata,* TP (Total phenolic (mg GAE/g)), TT (Total tannin (mg GAE/g)), NT (Non-tannin (mg GAE/g)), CT (Condensed tannin (mg CE/g)), PA (proanthocyanidin (mg LE)), GT (gallotannin (mg GAE/g)), FT (total flavonoid (mg QE/g)), FLL (flavonol (mg QE/g)).

The phenolic constituents of the crude extract of *Maytenus* species evaluated in this study varied widely as presented in Figure 3. The total phenolic content of the extracts ranged from 112.41 ± 1.51 to 197.44 ± 2.68 mg GAE/g plant material while the total tannin content ranged from 81.25 ± 2.66 to 1431.49 mg GAE/g plant material. *M. senegalensis* contained the highest content of total phenolics (197.44 ± 2.68 mg GAE/g dried plant material), total tannin (143.10 ± 1.49 mg GAE/g dried plant material), condensed tannin (35.29 ± 0.58), and proanthocyanidin (54.33 ± 2.01 mg. LE/g plant material) while *M. procumbens* contained the highest level of gallotannin (12.59 ± 3.80 mg GAE/g dried plant material) and flavonol (17.85 ± 0.19 mg QE/g plant material). The flavonoid content was however highest in *M. peduncularis* (77.45 ± 1.43 mg QE/g plant material). The enrichment of phenolic constituents in the extracts depends on the extracting solvent and the process of simultaneous extraction with acidified acetone solution and fractionation with n-hexane was effective in this regard. The hexane probably removed all components such as chlorophyll, waxes and terpenoids from the water fraction efficiently. The statistical analyses of the phenolic constituents indicated the presence of a good correlation between DPPH radical scavenging ability and total phenolic (R^2^ = 0.8826), non-tannin phenolic (R^2^ = 0.7110), total tannin (R^2^ = 0.9241) and proanthocyanidin (R^2^ = 0.6995) content. The same correlation was observed between the ABTS radical scavenging ability and total phenolic (R^2^ = 0.8768), non-tannin phenolic (R^2^ = 0.7016), total tannin (R^2^ = 0.9189) and proanthocyanidin (R^2^ = 0.6792) content. On the other hand, a low correlation was obtained between gallotannin (R^2^ = 0.2166), flavonoid (R^2^ = 0.4555) and flavonol (R^2^ = 0.3268) content and DPPH radical scavenging ability. A similar poor correlation was obtained between ABTS radical scavenging activity and gallotannin (R^2^ = 0.1997, flavonoid (R^2^ = 0.4632) and flavonol (R^2^ = 0.3014) content.

### Soybean 15-lipoxygenase inhibition

**Figure 4:**
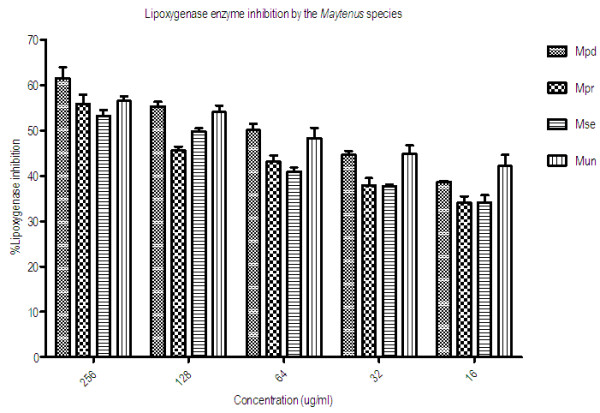
**Concentration-dependent percentage inhibition of soybean 15-lipoxygenase enzyme.** Mpd = *Maytenus peduncularis*, Mpr = *Maytenus procumbens*, Mse = *Maytenus senegalensis*, Mun = *Maytenus undata.*

The extracts of the four *Maytenus* species tested for *in vitro* inhibition of the enzyme soybean 15-lipoxygenase had varying activity presented as LL_50_ and percentage inhibitions in Table 1 and Figure 4 respectively. The most active extract was obtained from *M. peduncularis* with LL_50_ value of 44.16 ± 5.60 μg/ml followed by *M. undata* with LL_50_ of 50.92 ± 25.70 μg/ml. The activity of the extracts was concentration-dependent and the percentage inhibition ranged from 61.55 ± 4.19 μg/ml in *M. peduncularis* to 53.18 ± 2.30 μg/ml in *M. senegalensis* at the highest concentration of 256 μg/ml and 42.15 ± 4.29 μg/ml in *M. peduncularis* to 34.63 ± 2.55 μg/ml in *M. procumbens* at the lowest concentration of 16 μg/ml of the extracts.

## Discussion

Several therapeutic procedures have been used in orthodox medicine to treat diarrhoea symptoms, and associated complications. These include administration of oral rehydration therapy (ORT) as supportive therapy, use of antimicrobial drugs, anti-diarrhoeal agents (spasmolytic, motility, pro-absorptive or antisecretory) as symptomatic therapy and the use of probiotics (valuable in the treatment of rotavirus infections and post antibiotic diarrhoea) [54]. In traditional medicine, medicinal plants that contain a wide range of phytochemicals may exert an effect on more than one of the aspects listed above. The synergistic effects of the various medicinal properties may be responsible for the efficacy of plant preparations as antidiarrhoeal agents. However, the usefulness of medicinal plants in curing diarrhoea depends on the use of appropriate extractants. Two extractants such as water (decoction or infusion) and less frequently alcoholic solutions (tinctures) are mostly used in traditional medicines. Of equal importance is the dosage of the medicinal plant preparation administered to patients as applicable in conventional medicine.

Low MIC values indicate potentially high efficacy of the extracts as antimicrobial agents [55,56]. In our laboratory, crude extracts and fractions with an MIC <100 μg/ml are usually regarded as pharmacologically interesting and promising material for further studies.

The fractionation procedure used in this study potentiated the antimicrobial activities of the leaf extracts in the hexane and dichloromethane fractions with MICs ranging from 39 to 156 μg/ml against all the organisms tested. The intermediate polarity fraction of ethyl acetate and butanol fraction had moderate antimicrobial activities with MICs ranging from 312 to 2500 μg/ml. Crude extracts and water fractions had moderate to poor antimicrobial activities with MICs ranging from 312 to 2500 μg/ml. However, some fractions had no microbial growth inhibition at the highest concentration of 2500 μg/ml used in this study.

Neither Gram negative nor Gram positive bacteria were highly sensitive to the crude extracts and residual polar water fraction. This indicates that the antimicrobial compounds of *Maytenus* extracts are non-polar in nature. Therefore, in the treatment of infectious GIT including diarrhoea with leaf extracts of these plant species, the use of aqueous decoctions or infusions (polar extracts) which is the practice in traditional medicine may not be effective except where other antidiarrhoeal mechanisms are involved or if some nonpolar compounds were dissolved by microbial action during storage of concoctions. Absence of good antimicrobial activity from crude extracts and water fractions of all the four plants however, does not rule out the presence of such activity, but the phytoconstituents may be occurring in low ineffective concentrations. Many of the extracts and fractions were not highly active against microbes tested in this work. However, they may still be useful against infectious diseases since other mechanisms in healing process involving immuno-stimulatory [57,58] and toxin-deactivating activities [59], molecular changes of compound from inactive to active may take place during the digestive process, and uptake and metabolism. Some antibiotics, such as macrolides, sulfonamides and tetracyclines, exert their pharmacological effect by inhibiting the logarithmic growth of the bacterial organism, letting the immune system fight the infection and relieving the symptoms [60]. The pharmacological effect is not always related either to a bactericidal or to a bacteriostatic effect, but could be due to the inactivation of bacterial virulence factors.

Antimicrobial, anti-inflammatory and cytotoxicity of ethanol root extracts of *M. senegalensis* have been reported [24,25]. Maytenonic acid (3-oxofriedelan-20α-oic acid) (antibacterial activity against *Bacillus subtilus* (98 μg/ml), *Escherichia coli* (98 μg/ml), *Klebsiella pneumonia* (98 μg/ml) and *Staphylococcus aureus* (195 μg/ml)) [61] and pristimerin (antiplasmodial) [62] have been isolated from methanol root extract of *M. senegalensis.* Though this study did not establish the anti-infectious effect of leaf extracts from *Maytenus* species against all multitudes of microbes involve in GIT disorders, but they have demonstrated good potential as source of novel antimicrobial agents for the treatment of intestinal bacterial and fungal infections.

Oxidative stress and inflammatory damage of the GIT play an important role in initiating and maintaining diarrhoea symptoms. Oxidative species react with the mucosal epithelial membrane phospholipids to initiate and propagate the lipid peroxidation (LPO) process causing intestinal injury and cellular malfunction [63]. The products of lipid peroxidation include malondialdehydes, 4-hydroxyl-2, 3-nonenal, 4-hydroxy-2-hexenal and acrolein are cytotoxic [64]. In addition, the effects of LPO are also involved in the release of inflammatory mediators like cytokines, eicosanoids and generation of more reactive species [65]. Oxidative species such as hypochlorous acid, chloramine and some inflammatory mediators like prostaglandin E act directly as secretagogues in secretory or inflammatory diarrhoea [66]. An injured gastrointestinal tract (GIT) from lipid peroxidation also results in intestinal digestion and absorption disorders causing osmotic diarrhoea. Medicinal plants with good antioxidative or anti-inflammatory activities can be used as supportive therapy in diarrhoeal disease to reduce the oxidative damage to tissue cells.

The hexane, dichloromethane and water fractions had low antioxidant activities suggesting that their chemical components are extremely weak hydrogen or electron donors. However, antioxidant activities of the four plants were potentiated in the crude extracts, ethyl acetate and butanol fractions. Differences in antioxidant activity between the various solvents may due to variation in polyphenol concentration extracted. Different solvents have different degrees of solubility depending on their polarity [67].

Acetone and ethanol leaf extracts of *M. procumbens* have been reported to have anticancer and antioxidant properties. Two bioactive compounds, namely, 30-hydroxy-11a-methoxy-18b-olean-12-en-3-one and asiatic acid have been isolated from the extract [22]. Radical scavenging activity potential of *Maytenus* species observed in this study was a promising outcome for possible control of many oxidative stress-related diseases. The leaf extracts from the four plants worked better as antiradical agents than as lipid peroxidation inhibitors, although high antioxidant activity was obtained using all the assays.

Almost all diseases are characterized by the inflammation and pain in response to different conditions like injuries and infection indicate the presence of cyclooxygenase and lipoxygenase (LOX). The lipoxygenase (LOX) inhibitory potential of the extracts of *Maytenus* species was determined using linoleic acid peroxidation to hydroperoxy linoleic acid (leukotriene (LT) catalysed by soybean 15-lipoxygenase enzymes. Although, the enzyme used is from a plant source, the activities are similar to arachidonic acid and mammalian 15-LOX as substrates. However, there can be some differences in primary structure and mechanistic pathways. LOXs are sensitive to antioxidants, and the common mechanism of action is inhibition of lipid hydroperoxide formation due to scavenging of lipid oxy- or 9-lipid peroxy-radicals formed in the course of enzyme peroxidation. This limits the availability of lipid hydroperoxide substrate necessary for the catalytic cycle of LOX [68]. Another mode of LOX inhibition is attained via chelation of its non-haem bound iron or by reduction of its ferric form [68].

Antimicrobial, anti-inflammatory, antioxidant and antimalarial activity of ethanol leaf extracts of *Maytenus undata* (Thunb.) have been reported [23,69]. Phytochemical studies of the ethanol leaf extract of *Maytenus* have led to isolation and characterization of a number of bioactive compounds with antistaphylococcal properties. The compounds include 3-oxo-11α-methoxyolean-12-ene-30-oic acid, 3-oxo-11α-hydroxyolean-12-ene-30-oic acid, 3-oxo-olean-9(11),12-diene-30-oic acid, 3,4-seco-olean-4(23),12-diene-3,29-dioic acid (20-epikoetjapic acid), 3,11-dioxoolean-12-ene-30-oic acid (3-oxo-18β-glycyrrhetinic acid), koetjapic acid, 12-oleanene artifact 3-oxo-11α-ethoxyolean-12-ene-30-oic acid [69]. The MICs of the compounds varied between 3.125-6.25 μg/ml against methicillin-resistant *S. aureus, S. aureus* and *P. aeruginosa*[69].

Leukotrienes and other inflammatory mediators derived from LOX oxidation of polyunsaturated fatty acids (PUFA) are involved in intestinal smooth muscle lining contractions in diarrhoea and allergic reactions. The concentration-dependent inhibition of LOX by the phenolic-enriched *Maytenus* extracts indicated possible modulation of LT synthesis and therefore, the extracts can assist against diarrhoea through this mechanism.

For medicinal plant extracts to be useful in clinical application, the preparation should preferably be selectively toxic to the targeted organism or interfere directly with a specific reaction pathway. There should be no serious effect on the host cell or interference with normal physiological metabolism. The phenolic-rich *Maytenus* species extracts tested in this study were relatively non-toxic compared to the positive control (berberine chloride). However, *in vivo* acute toxicity studies are necessary to confirm the safety level of the extracts as *in vitro* assay results do not necessarily translate to *in vivo* activity. Effects of long term usage of the extracts such as mutagenicity and genotoxicity also need to be determined.

Phytochemical analyses of the crude extracts of the *Maytenus species* by other authors revealed the presence of varied quantities of phenolic compounds such as proanthocyanidins, flavonoids, flavonols and gallotannins. This group of compounds are important pharmacologically, being the antioxidant derivatives of medicinal and dietary plants. They also have other pharmacological activities such as antimicrobial (microbicidal or microbiostatic), anti-inflammatory, antiviral, antidiarrhoeal [70], and immune modulatory activities. The phenolic-enriched crude extracts and the polar fractions of the *Maytenus* species tested in this study had good antioxidative activities in one or more of the antioxidant assays relevant to inflammation and diarrhoeal diseases. Therefore, the antioxidant activity of the polar fractions in combination with the potentiated antimicrobial activity of the non-polar fractions of the four *Maytenus* species may be useful as therapeutic agents against the broad spectrum of diarrhoeal mechanisms. The results of this study, therefore, give a scientific basis to the use of these plants in traditional medicine as antidiarrhoeal therapy.

## Conclusion

The hexane, dichloromethane and ethyl acetate fractions had substantial antimicrobial activities against standard strains of diarrhoeagenic pathogens. The antimicrobial activities of these fractions coupled with the good antioxidative activities of the polar fractions in different oxidative mechanisms, especially diarrhoeic relevant lipid peroxidation and hydroxyl radical inhibition may provide relief in diarrhoea. These activities therefore provide a scientific base for the use of these plant species as antidiarrhoeal agents. The cytotoxicity assay results indicated that the extracts are relatively safe, but dosage still needs to be monitored in order to not exceed the toxicity level of the extracts. Other antidiarrhoeal mechanisms such as spasmolytic, antisecretory, antimotility and pro-absorption properties of the extracts may also have to be investigated so as to establish the actual mechanisms of action.

In many cases the ethyl acetate fraction had the highest antibacterial and antioxidant activities. If the safety of these fractions can be confirmed and there are no bioavailability or stability problems, these fractions may be developed into potentially useful therapeutic products to treat diarrhoea in production animals.

## Abbreviations

ABTS: 2,2’ azinobis(3-ethylbenzthiazoline-6-sulphonic acid); ATCC: American type culture collection; CE: Catechin equivalent; CFU: Colony-forming units; CD4+: Count of CD4 expressing helper T; DMSO: Dimethylsulphoxide; DPPH: 1, 1-Diphenyl-2-picrylhydrazyl; GAE: Gallic acid equivalent; GIT: gastrointestinal tract; IC50: Inhibitory concentration for 50% of maximum response; INT: p-iodonitrotetrazolium violet; LL50: Lipoxygenase inhibitory concentration for 50% of maximum response; LE: Leucoproanthocyanidin equivalent; LOX: Lipoxygenase; LPO: Lipid peroxidation; MIC: Minimum inhibitory concentration; MTT: 3-(4, 5-dimethylthiazol-2-yl)-2, 5 diphenyltetrazolium bromide; OH: Hydroxyl; PVPP: polyvinylpyrrolidone; QE: Quercetin equivalent

## Competing interests

The authors declare that they have no competing interests.

## Authors’ contributions

ASA participated in the study design, performed the experiments and data entry, analysed the results, and draft the manuscript. LJM participated in the study design, performed the cytotoxicity experiments and data entry, and critically reviewed the manuscript. JNE conceived and participated in the design of the study, reviewed the results, and critically reviewed the manuscript. All authors read and approved the final manuscript.

## Pre-publication history

The pre-publication history for this paper can be accessed here:

http://www.biomedcentral.com/1472-6882/13/100/prepub
